# Innate Immune Response to *Streptococcus pyogenes* Depends on the Combined Activation of TLR13 and TLR2

**DOI:** 10.1371/journal.pone.0119727

**Published:** 2015-03-10

**Authors:** Christina Fieber, Marton Janos, Tina Koestler, Nina Gratz, Xiao-Dong Li, Virginia Castiglia, Marion Aberle, Martina Sauert, Mareike Wegner, Lena Alexopoulou, Carsten J. Kirschning, Zhijian J. Chen, Arndt von Haeseler, Pavel Kovarik

**Affiliations:** 1 Max F. Perutz Laboratories, University of Vienna, Vienna, Austria; 2 Center for Integrative Bioinformatics Vienna, Max F. Perutz Laboratories, University of Vienna, Medical University of Vienna, Vienna, Austria; 3 Universitätsklinikum Freiburg, Universitäts-Hautklinik, Freiburg, Germany; 4 Centre d'Immunologie de Marseille-Luminy (CIML), Aix-Marseille Université UM 2, Marseille, France; 5 Institute of Medical Microbiology, University of Duisburg-Essen, Essen, Germany; 6 Howard Hughes Medical Institute, Department of Molecular Biology, University of Texas Southwestern Medical Center, Dallas, Texas, United States of America; 7 Bioinformatics and Computational Biology, Faculty of Computer Science, University of Vienna, Vienna, Austria; McGill University, CANADA

## Abstract

Innate immune recognition of the major human-specific Gram-positive pathogen *Streptococcus pyogenes* is not understood. Here we show that mice employ Toll-like receptor (TLR) 2- and TLR13-mediated recognition of *S*. *pyogenes*. These TLR pathways are non-redundant in the in vivo context of animal infection, but are largely redundant in vitro, as only inactivation of both of them abolishes inflammatory cytokine production by macrophages and dendritic cells infected with *S*. *pyogenes*. Mechanistically, *S*. *pyogenes* is initially recognized in a phagocytosis-independent manner by TLR2 and subsequently by TLR13 upon internalization. We show that the TLR13 response is specifically triggered by *S*. *pyogenes* rRNA and that *Tlr13* ^*−/−*^ cells respond to *S*. *pyogenes* infection solely by engagement of TLR2. TLR13 is absent from humans and, remarkably, we find no equivalent route for *S*. *pyogenes* RNA recognition in human macrophages. Phylogenetic analysis reveals that TLR13 occurs in all kingdoms but only in few mammals, including mice and rats, which are naturally resistant against *S*. *pyogenes*. Our study establishes that the dissimilar expression of TLR13 in mice and humans has functional consequences for recognition of *S*. *pyogenes* in these organisms.

## Introduction

Several receptor families are employed by innate immune cells to detect infecting agents, Toll-like receptors (TLRs) being the most prominent members. Mammals possess several TLRs which have most likely evolved by gene duplication and exon shuffling from an ancestral gene early in metazoan evolution [1]. TLRs recognize specific pathogen-associated molecular patterns (PAMPs) which are common to different pathogen species. *Streptococcus pyogenes*, also called Group A Streptococcus, is an important Gram-positive human pathogen yet its recognition by innate immune cells remains unknown [2,3]. *S*. *pyogenes* causes a broad range of mostly self-limiting diseases including pharyngitis (strep throat), scarlet fever or impetigo [4,5]. It may also cause invasive and life-threatening infections such as necrotizing fasciitis and toxic shock with ∼30% mortality rate. *S*. *pyogenes* accounts for over 700 million mild and more than 650,000 severe invasive infections worldwide annually [6]. Together with *S*. *pneumoniae*, *S*. *pyogenes* is one of the most frequently found co-infecting bacteria in specimens of the 1918 flu pandemics and in patients of the recent H1N1 flu outbreak [7].

The exceptionally large variety of *S*. *pyogenes*-related infectious diseases is caused in part by variations of virulence factor armament of *S*. *pyogenes* strains and in part by the genetic makeup of the host [8,9]. On the host site, animal studies demonstrated that innate immune cells, most notably macrophages, dendritic cells and neutrophils, play an essential role in defense during subcutaneous infection, a model of invasive *S*. *pyogenes* infection [10–13].

Despite the importance of the innate immune system for host defense, the TLRs and PAMPs involved in functional recognition of *S*. *pyogenes* are not defined. We and others have shown that *S*. *pyogenes*-induced production of inflammatory cytokines, including TNF and IL-6, by murine bone marrow-derived macrophages (BMDMs; bone marrow cells differentiated in the presence of CSF1) and conventional dendritic cells (cDCs; bone marrow cells differentiated in the presence of GM-CSF) is entirely dependent on the signaling adaptor MyD88. Consistently, MyD88 is required for survival of mice during *S*. *pyogenes* infection [14]. The TLRs triggering the protective innate immune response are not known. Studies by us and others demonstrated that *S*. *pyogenes* induces cytokine production in the absence of the MyD88-dependent TLR2, TLR4 and TLR9 [13,15]. A 13 nucleotide long sequence of bacterial 23S rRNA has been recently demonstrated to act as PAMP recognized by the TLR13 in murine cells [16–18]. TLR13, whose ligand has long remained unknown, is located in endosomes and similarly to other endosomal TLRs (TLR3, TLR7, TLR9) requires Unc93b1, a COPII vesicle membrane protein, for trafficking to endosomes [19,20]. Deletion of Unc93b1 abolishes responses of cells to ligands sensed by endosomal TLRs including TLR13 [19,20]. It remains unknown whether recognition of bacterial rRNA by TLR13 regulates the host defense in mice. Importantly, it is not understood how human immune cells recognize *S*. *pyogenes* and whether *S*. *pyogenes* RNA plays a role in this process.

Here we report that *S*. *pyogenes* infection of mouse BMDMs and cDCs triggers both TLR2 and TLR13 pathways. Both pathways are to large part redundant in vitro: the TLR2 pathway becomes apparent only in *Tlr13* ^*−/−*^ cells thereby explaining the lack of evidence for a role of TLR2 in previous studies. The TLR13 pathway is activated by *S*. *pyogenes* rRNA and is dependent on phagocytosis and endosomal recognition. Consistently, we find that Unc93b1 plays an essential role in cytokine induction by *S*. *pyogenes* RNA. Unexpectedly, the TLR2 and the endosomal TLR recognition pathway are not redundant in vivo: mice deficient in either of these pathways display an increased susceptibility to *S*. *pyogenes* infection. These data show that a protective immune reaction is mounted only by triggering both pathways. Humans lack TLR13 and we find that human innate immune cells are not capable of inducing TNF and IL-8 in response to *S*. *pyogenes* RNA. We demonstrate that human cells sense *S*. *pyogenes* through TLR2. However, human primary macrophages produce TNF and IL-8 upon *S*. *pyogenes* infection also under conditions of antibody-mediated TLR2 inhibition indicating that a TLR2-independent sensing pathway operates in these cells. Phylogenetic analysis reveals a remarkable lack of TLR13 in primates including humans as opposed to mice and rats, which possess highly conserved *Tlr13* genes and are naturally resistant against *S*. *pyogenes*. As exemplified by our study, the lack of TLR13 in humans results in the lack of TLR-mediated recognition of *S*. *pyogenes* rRNA.

## Materials and Methods

### Ethics statement

All animal experiments were carried out in accordance with the Austrian law for animal care (GZ 680 205/67-BrGt/2003). Animal experiments were authorized through the licenses BMWF-66.009/0031-II/10b/2008 and BMWF-66.006/0006-II/3b/2013, issued by the Austrian Ministry of Science to PK.

### Bacterial cultures

The *Streptococcus pyogenes* serotype M1 strains ISS3348 (provided by Roberta Creti, Instituto Superiore di Sanita, Italy) was used as described previously [21].

### Mice

*Tlr2* ^*−/−*^, *Tlr3* ^*−/−*^, *Tlr7* ^*−/−*^, *Tlr8* ^*−/−*^, *Tlr379* triple knockout, *Trif* ^*−/−*^, *Tlr13* ^*−/−*^, *Unc93b1* ^*−/−*^, and *Unc93b1Tlr2* double knockout, *MyD88* ^*−/−*^ mice were on C57BL/6 background and housed under specific pathogen-free conditions. C57BL/6 wild type (WT) mice were purchased from Charles River Laboratories. Age- and sex-matched 7–12 weeks old mice were used for experiments.

### Cell culture

Primary bone marrow derived macrophages (BMDMs) and conventional dendritic cells (cDCs) were obtained from the femur and tibia bone marrow of 7–10 weeks old mice and stimulated as described previously [15,21]. Human Embryonic Kidney 293 cells (HEK) cells were cultivated in DMEM with 10% FCS. HEK293XL cells stably expressing either human TLR7 or TLR8 were obtained from Invivogen, and HEK cells stably expressing human TLR3 were generated by stable transfection with the vector pUNO-HA bearing human TLR3 (Invivogen). THP-1 cells were cultivated in Roswell Park Memorial Institute medium (RPMI) with 10% FCS. THP-1 cells were differentiated using 10 nM PMA in RPMI with 10% FCS for 24 hours followed by cultivation for additional 48 hours in RPMI with 10% FCS without PMA. THP-1 cells stably expressing TLR13 were generated by lentiviral transduction. HA-tagged mouse TLR13 was cloned from pUNO1-mTLR13-HA3x (Invivogen) into pCDH-CMV-MCS-EF1-puro (System Biosciences) and packaged in HEK293T cells using pMDLg/pRRE (Addgene#12251), pCMV-VSV-G (Addgene #8454) and pRSV-Rev (Addgene #12253). Transduced cells were selected with 1.5 μg/ml puromycin followed by clonal expansion and screening for clones positive for expression of the HA epitope-tagged TLR13. Human peripheral blood mononuclear cells (PBMC) were isolated by Ficoll-Paque (GE Healthcare) density centrifugation from buffy coats (purchased from Red Cross, Vienna). To differentiate PBMC into monocyte-derived macrophages approximately 1–2x10^8^ cells were plated in RPMI 1640 media with 2 mM L-glutamine containing 7.5% heat-inactivated (HI) autologous human fibrin-depleted plasma and antibiotics (MoAM; monocyte adhesion media) in 15 cm round dishes for 45–90 min. Non-adherent cells were removed by gently washing the dish with warm media, fresh MoAM was added and the cells were left in an incubator at 37°C with 5% CO2 overnight. The next day, the monocytes were detached with PBS containing 5 mM EDTA, counted and plated in X-Vivo 10 (Lonza, 04-743Q) supplemented with 1% HI autologous human fibrin-depleted plasma, 2 mM glutamine and antibiotics. The experiment was performed on day 7 to 8.

### Experimental model of *S*. *pyogenes* infection

Standard model of subcutaneous *S*. *pyogenes i*nfection was used as described previously [21]. Survival curves were analyzed by the Logrank Test using GraphPad Prism 4 (GraphPad software, San Diego, USA). All data are presented as mean SD. Comparison between two groups was performed using t-test. P values ≤0.05 were considered as significant.

### *S*. *pyogenes* infection in vitro

For infection assays, cells were seeded on 6 cm dishes (1x10^6^ cells/dish) or 12-well plates (2–3x10^5^ cells/well for human primary macrophages) in media without antibiotics. Cells were then infected as described previously with some minor changes [21]. Briefly, cells were infected with *S*. *pyogenes* at a multiplicity of infection (MOI) of 50 or 100. After 30 min of incubation at 37°C, bacteria were killed by adding 60 μg/ml penicillin to the media. Infections under conditions of blocked phagocytosis were performed by pre-treatment of cells with cytochalasin D (50 μM; Sigma) 30 min prior to infection with *S*. *pyogenes*. For TLR neutralization, antibodies against human TLR2 (5 μg/ml; Invivogen, maba2-htlr2) and control IgG antibodies (5 μg/ml; Invivogen, maba2-ctrl), or antibodies against mouse TLR2 (5 μg/ml; eBioscience, 16-9024-83) and control IgG (5 μg/ml; eBioscience, 14-4714) were used 30 min prior to infection.

### *S*. *pyogenes* extracts

*S*. *pyogenes* extracts treated with proteinase K, DNase or RNase were generated as described previously [21].

### *S*. *pyogenes* RNA and DNA fractions

Isolation of bacterial RNA and DNA was performed according to standard techniques, with some modifications [22]. Briefly, for RNA isolation bacteria were grown to mid-log phase and lysed using 2% SDS and 1 mg/ml proteinase K (Sigma-Aldrich). For isolation of RNA, Trizol LS reagent (Invitrogen) was used. RNA was treated with DNase (TURBO DNA-free; Ambion). For RNA-free samples RNA was treated with RNase A (Roche, 10 mg/ml, heat-inactivated) for 30 min at 37°C followed by addition of EDTA (pH 8, 2.5 mM) and further incubation for 10 min at 70°C. For control, sample containing only H_2_O (RNase control) was treated in the same way. mRNA fraction was obtained using the MICROBExpress Kit (Ambion). Briefly, after the Trizol step of the total bacterial RNA isolation fragments shorter than 200 nucleotides were removed from total RNA using miRNeasy Mini Kit (Qiagen). mRNA was then isolated according to the MICROBExpress Kit protocol. Bacterial rRNA was eluted from the MICROBExpress Kit beads by adding TE buffer and incubating for 10 min at 70°C. Bacterial mRNA isolated with the MICROBExpress Kit contained 0.3% bacterial rRNA as assessed with qPCR analysis. Pure bacterial mRNA was obtained from total RNA using the Ribo-Zero rRNA Removal Kit (Epicenter) which yields mRNA without contaminating rRNA, but does not allow elution of rRNA. For DNA preparation, over-night cultures of bacteria were harvested, resuspended in TE buffer (10 mM Tris-HCl pH 8, 1 mM EDTA) and treated with lysozyme (Sigma, 2.5 mg/ml) for 30 min at 37°C. Subsequently, RNase A (10 mg/ml), 20% SDS and 0.5 M EDTA were added. After incubation for 40 min at 37°C, 20 mg/ml proteinase K was added, and the samples were further incubated for 30 min at 37°C. DNA was isolated using phenol:chloroform:isoamylalcohol (Sigma-Aldrich). Isolated bacterial DNA was treated with RNase A (10 mg/ml) for 30 min at 37°C. Adherent macrophages and HEK cells were seeded (1x10^6^ cells per 6 cm dish) the day prior to transfection, non-adherent cDCs and THP-1 cells (1x10^6^ cells per 6 cm dish) were seeded 2 hours prior to transfection, PMA-stimulated THP-1 cells were seeded 3 days prior to transfection (1x10^6^ cells per 6 cm dish) with 1 to 5 μg RNA or DNA using DOTAP. The transfection mixture was added to dishes containing 1.5 ml media without antibiotics.

### Transfection and stimulation of cells

For one dish or well, 10 μl of bacterial extracts (containing 5 μg of nucleic acids), or 1 to 5 μg of purified RNA or DNA were used to form complexes with 30 μl DOTAP (Roche) as described previously [21]. The TLR3 ligand poly(I:C) (5 μg, Amersham), the TLR13 ligand (RNA oligoribonucleotide SA19 with the sequence 5’-GGACGGAAAGACCCCGUGG-3’ [16], 1 μg or 5 μg) and the poor TLR13 ligand (RNA oligoribonucleotide mut-SA19 with the sequence 5’-GGACGGGAAGACCCCGUGG-3’ [16], 1 μg) were transfected into cells using DOTAP. The TLR7 and TLR8 ligand R848 (Invivogen) was used at 5 μg/ml, LPS (from *E*.*coli*, Sigma) was used at 10 ng/ml and LTA (Invivogen) was used at 5 μg/ml.

### Fluorescence microscopy

Fluorescence microscopy was performed as described previously [21].

### Cytokine measurement

Cytokines in supernatants of stimulated cells were measured using DuoSET ELISA kits (R&D Systems) for mouse and human TNF, mouse IL-6 and human IL-8.

### Quantitative RT-PCR (qRT-PCR)

qRT-PCR was performed as described previously [21] using Quantitec Primer Assays (QIAGEN) and the SYBR Green (Promega) detection on the Realplex Mastercycler (Eppendorf). For normalization, the mouse *Hprt* primers mHPRT-fwd 5′-GGATTTGAATCACGTTTGTGTCAT-3′, and mHPRT-rev 5′-ACACCTGCTAATTTTACTGGCAA-3′; and the human *HPRT* primers hHPRT- fwd 5- TGT GTG CTC AAG GGG GGC-3’ and hHPRT-rev 5’-CGT GGG GTC CTT TTC ACC-3’ were used.

### Identification of predicted TLR13 orthologs

Mouse TLR13 protein sequence was used for searching the OMA Browser (http://omabrowser.org) [23] which contains about 1,320 species. To account for species not present in the OMA Browser we additionally performed a standard NCBI Blast search (http://blast.ncbi.nlm.nih.gov) [24] against the non-redundant protein sequences [25]. The OMA Browse hits amended with NCBI Blast hits were further analyzed using FACT (Feature Architecture Comparison Tool; http://www.cibiv.at/FACT) [26] to identify those sequences which exhibit similar feature architecture as the query protein (mouse TLR13). If the architecture was similar, then the table entry was regarded as predicted ortholog. Thus, the predicted orthologs are similar in sequence and in their feature architecture and can be regarded to a high degree of confidence as true orthologs.

### Phylogenetic analysis

The evolutionary relationships among TLR13 proteins in species and TLR repertoires in humans and mice were constructed using alignment with mafft with the linsi option [27], and the tree was reconstructed with iqtree (-m TEST −b 100) [28].

### GenBank accession numbers

*Tnf*: NM_013693, *TNF*: NM_000594, *Il6*: NM_031168, *Il8*: NM_000584, *MyD88*: NM_010851, *Tlr2*: NM_011905, *Tlr3*: NM_126166, *TLR3*: NM_003265, *Tlr4*: NM_021297, *Tlr7*: NM_133211, *TLR7*: NM_016562, *Tlr8*: NM_133212, *TLR8*: NM_138636, Tlr13: NM_205820, *Trif*: NM_174989, *Unc93b1*: NM_019449, *Hprt*: NM_013556, *HPRT*: NM_000194

## Results

### *S*. *pyogenes* rRNA activates mouse BMDMs and cDCs by triggering TLR13

**Fig 1:**
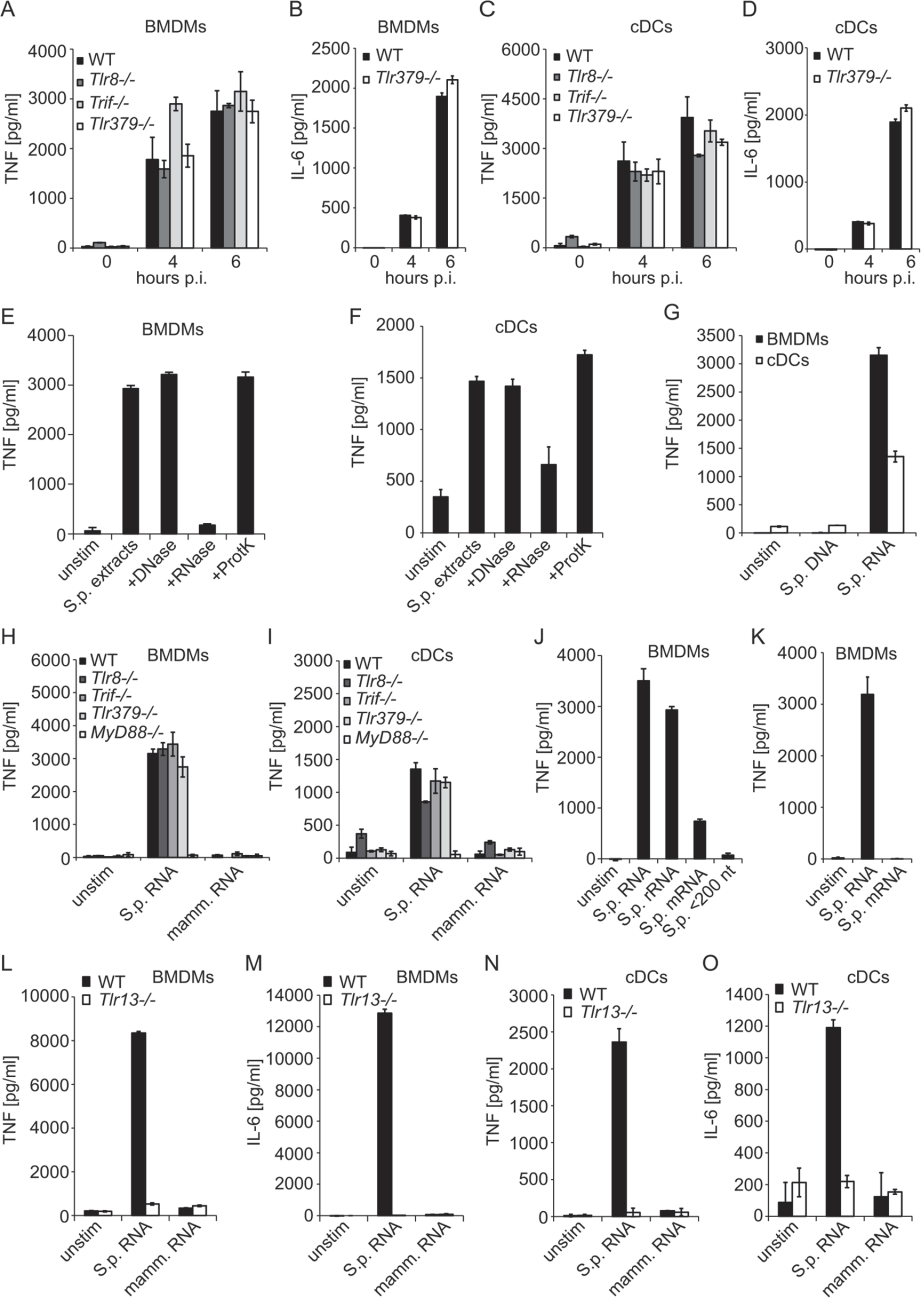
*S*. *pyogenes* rRNA induces cytokine production in a TLR13-dependent way and independently of TRIF, TLR3, TLR7, TLR8 and TLR9. BMDMs (A, B) or cDCs (C, D) from *Tlr8−/−*, *Trif−/−* or *Tlr379* triple-deficient mice as well as control mice (WT) were infected with *S*. *pyogenes* (MOI = 50) or left uninfected. At indicated time points, supernatants were collected and TNF (A, C) or IL-6 (B, D) release was measured by ELISA. (E, F) *S*. *pyogenes* cells were sonicated and the extracts were treated with either DNase I, RNase A, proteinase K, or left untreated (control extract). These extracts were delivered into BMDMs (E) or cDCs (F) using DOTAP. After stimulation for 6 h, supernatants were collected and TNF release was measured by ELISA. (G) RNA or DNA isolated from *S*. *pyogenes* was transfected into WT BMDMs or cDCs and after 6 h of stimulation supernatants were collected and TNF release was measured by ELISA. (H, I) BMDMs (H) and cDCs (I) from *Tlr8−/−*, *Trif−/−*, *MyD88−/−* and *Tlr379* triple-deficient mice as well as control mice (WT) were transfected with *S*. *pyogenes* or mammalian RNA or left untreated. Supernatants were collected after 6 h and TNF release was measured by ELISA. (J) *S*. *pyogenes* total RNA as well as the mRNA (isolated with the MICROBExpress Bacterial mRNA Enrichment Kit, contains 0.3% rRNA), rRNA and nt>200 fractions were used for transfection of BMDMs. TNF release was determined in supernatants of cells after 6 h of stimulation using ELISA. (K) *S*. *pyogenes* total RNA as well as the mRNA (isolated with the Ribo-Zero rRNA Removal Kit) were transfected into BMDMs. TNF release was measured 6 h after stimulation using ELISA. (L—O) BMDMs (L, M) and cDCs (N, O) from *Tlr13−/−* mice as well as control mice (WT) were transfected with *S*. *pyogenes* or mammalian RNA or left untreated. Supernatants were collected after 6 h and TNF (L, N) or IL-6 (M, O) release was measured by ELISA. Error bars in all panels represent SDs (n≥3).

*S*. *pyogenes* infection of BMDMs and cDCs induces a cytokine burst in a MyD88-dependent but TLR2-, TLR4- and TLR9-independent way [13,15]. We tested the role of other TLRs known to signal in MyD88-dependent manner. We included TLR3/TLR7/TLR9 (TLR379) triple-deficient cells to account for potential redundancy of these three nucleic acids-sensing TLRs, Trif-deficient cells to directly assess the downstream signaling adaptor of TLR3, and TLR8-deficient cells. BMDMs and cDCs lacking TLR8 or Trif as well as cells TLR379 triple-deficient cells responded to *S*. *pyogenes* infection by producing TNF and IL-6 in comparable amounts to wild type (WT) control cells (Fig. 1A and C). Similarly, induction of IL-6 was not impaired in TLR379 triple-deficient BMDMs and cDCs (Fig. 1B and D).

We have recently shown that *S*. *pyogenes* RNA induced IFN-β in cDCs, but not BMDMs [21]. *S*. *pyogenes* DNA was able to stimulate IFN-β in BMDMs but not cDCs [21]. Further, *S*. *pyogenes* RNA was shown to induce proinflammatory cytokines in cDCs [18]. To directly compare the stimulatory activity *S*. *pyogenes* nucleic acids in BMDMs and cDCs, we transfected *S*. *pyogenes* extracts treated with DNase I, RNase A, or proteinase K into cells using DOTAP which mediates delivery into endosomes but not cytosol [29]. With the exception of the RNase A-treated fraction, all were able to activate TNF production in both cell types (Fig. 1E and F) showing that *S*. *pyogenes* RNA but not DNA was functionally recognized. DNA purified from *S*. *pyogenes* did not stimulate TNF production in BMDMs and cDCs (Fig. 1G). Purified *S*. *pyogenes* RNA elicited responses in TLR8-, Trif-, and TLR379-deficient BMDMs and cDCs but not in *MyD88−/−* cells (Fig. 1H and I). Thus, stimulation of mouse innate immune cells with *S*. *pyogenes* RNA recapitulated responses to infection of cells with live *S*. *pyogenes*.

rRNA of several bacterial species was shown to stimulate TLR13 [16,17]. Total *S*. *pyogenes* RNA could stimulate TLR13 in cDCs [18]. To determine the stimulatory function of *S*. *pyogenes* RNA in more detail, we separated total *S*. *pyogenes* RNA into mRNA, 16S and 23S rRNA, and into a fraction containing RNA smaller than 200 nucleotides including tRNA and 5S rRNA. Stimulation of BMDMs with rRNA resulted in TNF induction similar to stimulation with total RNA (Fig. 1J). In contrast, RNA <200 nucleotides was not stimulatory and the mRNA fraction exhibited a strongly decreased ability to induce TNF (Fig. 1J). Detailed analysis revealed that the mRNA fraction contained 0.3% rRNA. To evaluate whether the contaminating rRNA in the mRNA fraction was responsible for the residual TNF-inducing activity we employed a different RNA separation method which allowed isolation of pure mRNA but not other RNA fractions. The rRNA-free mRNA was no longer able to induce TNF (Fig. 1K). Thus, rRNA is the sole cytokine-inducing component of *S*. *pyogenes* RNA in both BMDMs and cDCs suggesting TLR13-mediated recognition, as reported for 23S rRNA from other bacterial species [16,17]. We confirmed the role of TLR13 by using *Tlr13* ^*−/−*^ BMDMs and cDCs: *Tlr13* ^*−/−*^ cells were not responding to *S*. *pyogenes* RNA (Fig. 1L-O). In conclusion, TLR13 is a non-redundant immune receptor for sensing *S*. *pyogenes* rRNA by both BMDMs and cDCs.

### *S*. *pyogenes* infection triggers TLR2 and Unc93b1-dependent TLR13 responses

**Fig 2:**
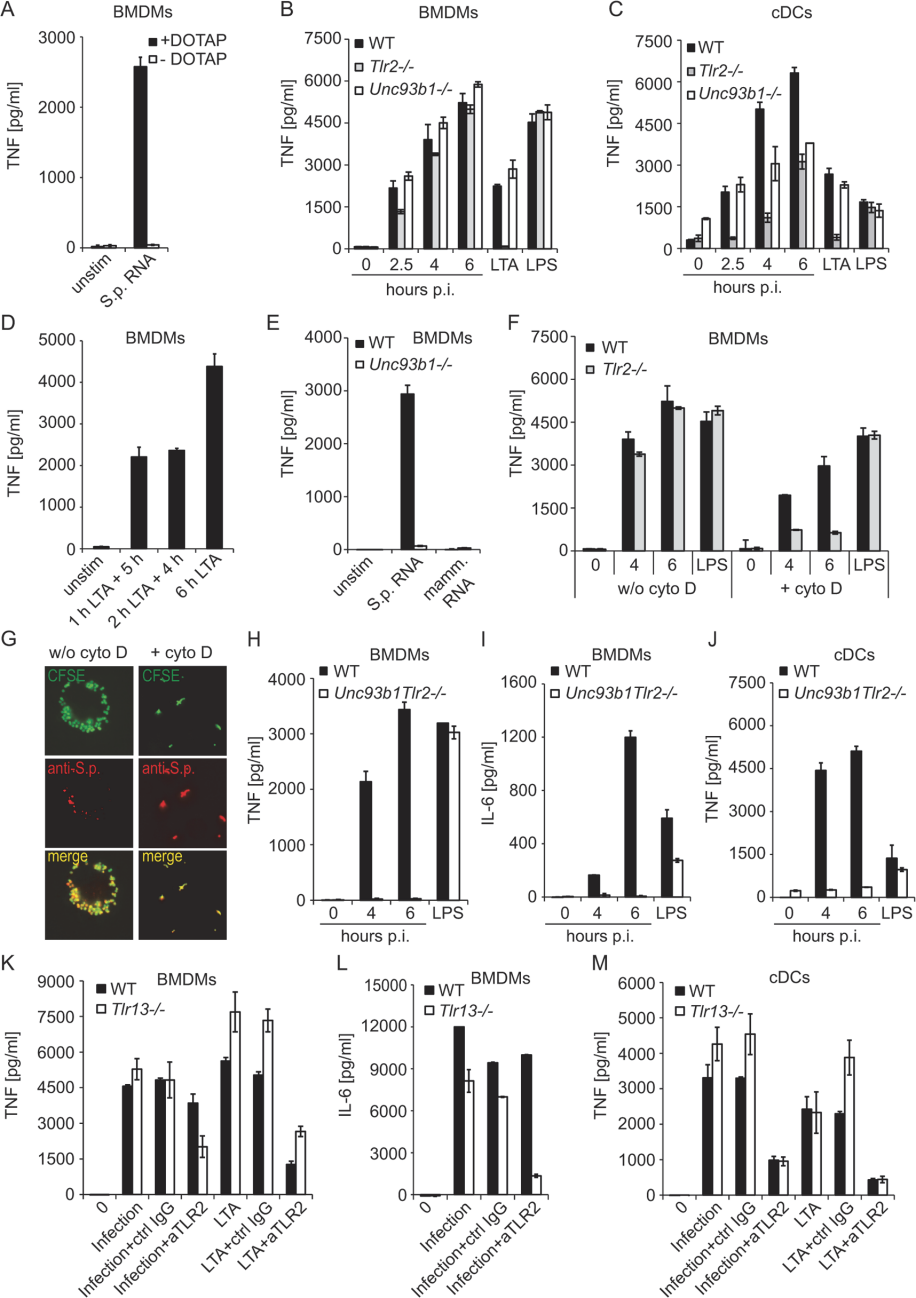
*S*. *pyogenes* is recognized by a combination of Tlr2- and Tlr13-mediated sensing. (A) *S*. *pyogenes* RNA was either added directly to the BMDMs or transfected into BMDMs using DOTAP. Supernatants were collected after 6 h and TNF release was measured by ELISA. (B, C) BMDMs (B) or cDCs (C) from *Tlr2−/−* and *Unc93b1−/−* mice as well as control mice (WT) were infected with *S*. *pyogenes* (MOI = 50) or left uninfected, or treated with LTA or LPS as a control. At indicated time points, supernatants were collected and TNF release was measured by ELISA. (D) BMDMs were stimulated for 1 or 2 h with LTA, and after medium change cells were incubated without LTA for additional 5 or 4 h, respectively, As a control, BMDMs were treated with LTA for 6 h. TNF was determined in the supernatants by ELISA. (E) BMDMs from *Unc93b1−/−* mice as well as control mice (WT) were transfected with streptococcal- or mammalian RNA. Supernatants were collected after 6 h and TNF release was measured by ELISA. (F) BMDMs from *Tlr2−/−* as well as control mice (WT) were pre-treated for 30 min with cytochalasin D or left untreated prior infection with *S*. *pyogenes* (MOI = 50). At indicated time points, supernatants were collected and TNF release was measured by ELISA. (G) BMDMs were pre-treated with cytochalasin D (right panels) or left untreated (left panels) before infection with CFSE-labeled (green) *S*. *pyogenes* (MOI = 50). After 4 h cells were fixed and stained with anti *S*. *pyogenes* antibody (anti-S.p.) for extracellular bacteria (red). Immunofluorescence image depicts CFSE labeled bacteria (both extra- and intracellular) in green, extracellular antibody-stained bacteria in red, and a merge of the two channels in showing extracellular bacteria in yellow and intracellular bacteria in green. (H—J) BMDMs (H, I) and cDCs (J) from *Unc93b1Tlr2* double-deficient mice as well as control mice (WT) were infected with *S*. *pyogenes* (MOI = 50) or left uninfected. At indicated time points, supernatants were collected and TNF (H, J) or IL-6 (I) release was measured by ELISA. LPS treatment (6 h) served as specificity control. (K—M) BMDMs (K, L) and cDCs (M) from *Tlr13−/−* as well as control mice (WT) were incubated for 30–45 min with anti-TLR2, control IgG or left untreated prior to infection with *S*. *pyogenes* (MOI = 100) or stimulation with LTA. Supernatants were collected 4 h after infection and TNF (K, M) or IL-6 (L) release was measured by ELISA. Error bars in all panels represent SDs (n≥3).

TLR13 has been localized to endosomes suggesting that the recognition of *S*. *pyogenes* RNA occurs after phagocytosis [30]. In agreement, exposure of cells to *S*. *pyogenes* RNA in the absence of DOTAP did not result in TNF induction (Fig. 2A). The endosomal TLR sorting chaperon Unc93b1 is required for signaling through endosomal TLRs [19,20,31]. Unexpectedly, *S*. *pyogenes* infection of *Unc93b1*-deficient BMDMs resulted in comparable TNF production as infection of WT cells (Fig. 2B). These findings implied that during infection with live bacteria, the endosomal pathway recognizing *S*. *pyogenes* RNA was redundant with another mode of *S*. *pyogenes* recognition. Deficiency in TLR2, which is involved in endocytosis-independent recognition of cell wall components from Gram-positive bacteria, resulted in slightly reduced cytokine production early after infection but at later time points the responses of TLR2-deficient and WT BMDMs were comparable (Fig. 2B), in agreement with previous studies [13,15]. The contribution of TLR2-depedent pathway was more apparent in cDCs but it became less visible with increasing time of infection (Fig. 2C). Lack of TLR2 strongly impaired cytokine production by cDCs in the very early phase (i.e. 2.5 h post infection), whereas this phase appeared normal in Unc93b1-deficient cells. Moreover, the TLR2-mediated TNF accumulation (i.e. TNF produced in Unc93b1-deficient cells) was increasing over time suggesting that cells were producing cytokines also at later time points, i.e. at the time when the Unc93b1 pathway was also becoming active. To test this, we stimulated solely the TLR2 pathway using LTA, and replaced the LTA-containing medium with medium without LTA 1 or 2 h after stimulation followed by additional incubation for 5 or 4 h, respectively. The supernatants of these cells contained comparable TNF amounts, but the TNF levels were lower than those produced by cells with permanent presence of LTA (i.e. cells without media change) (Fig. 2D). This experiment demonstrated that the cells were continuously producing TNF during the incubation period. The temporal profile of TNF production by *S*. *pyogenes*-infected cells (Fig. 2B and D) was consistent with a largely host cell surface-localized TLR2-dependent *S*. *pyogenes* recognition early in the infection and an endosome-dominated recognition in the later phase. The endosomal recognition was triggered by *S*. *pyogenes* RNA, as revealed by the lack of responses in Unc93b1-deficient cells stimulated with RNA (Fig. 2E).

To confirm that in the absence of phagocytosis the recognition of *S*. *pyogenes* proceeds exclusively in a TLR2-dependent way, we infected WT and TLR2-deficient BMDMs in the presence of the phagocytosis inhibitor cytochalasin D. The blockage of phagocytosis almost abolished responses of TLR2-deficient cells to *S*. *pyogenes* (Fig. 2F) and prevented *S*. *pyogenes* internalization (Fig. 2G). To further substantiate the essential role of TLR2 and the endosomal TLRs in sensing *S*. *pyogenes* we infected TLR2 and Unc93b1 double-deficient cells.Responses of these cells to *S*. *pyogenes* infection were lost (Fig. 2H-J). Since we showed that TLR13 was the only endosomal TLR capable of sensing *S*. *pyogenes* (Fig. 1), the results obtained using TLR2 and Unc93b1 double-deficient cells further strengthened the exclusive roles of TLR2 and TLR13 in responses to *S*. *pyogenes*. Finally, antibody-mediated blocking of TLR2 in TLR13-deficient cells resulted in strong reduction of cytokine production 4 h after infection, at the time of predominantly TLR2-mediated cell surface-localized *S*. *pyogenes* recognition (Fig. 2K-M). Together, these results demonstrate that TLR2 and TLR13 pathways play a leading role in recognition of live *S*. *pyogenes* by murine cells. These two pathways are largely redundant and only the inactivation of both of them abolishes sensing of *S*. *pyogenes* infection.

### Unc93b1 and TLR2 are required for defense against *S*. *pyogenes* in mice

**Fig 3:**
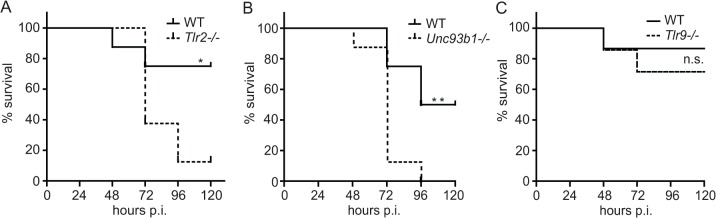
Entire activation of the immune responses through both Tlr2- and Unc93b1-dependent pathways is required for protective defense against *S*. *pyogenes* in mice. (**A**—**C**) Kaplan-Meier survival curves of C57BL/6 and *Tlr2−/−*, *Unc93b1−/−* (8 mice per genotype) or *Tlr9−/−* mice (14 mice per genotype) after subcutaneous infection with 1×10^8^ CFU of *S*. *pyogenes ISS3348*. Survival was monitored for 6 days. Significance: * = p<0.05; ** = p<0.01.

Considering the highly redundant functions of the TLR2- and Unc93b1-mediated sensing pathways in vitro (Fig. 2B-D), we asked whether these pathways were redundant during infection of mice. TLR2- or Unc93b1-deficient mice were subcutaneously inoculated with the *S*. *pyogenes* strain ISS-3348, an M1 serotype strain that is virulent in mice [21]. We observed an increased susceptibility of both TLR2- and Unc93b1-deficient animals to infection compared to WT controls (Fig. 3A-B). Thus, both the TLR2 and Unc93b1 pathways play essential and non-redundant roles in mouse defense against *S*. *pyogenes*. Consistent with the inability of S. pyogenes DNA to elicit TNF production in vitro (Fig. 1G), mice lacking the DNA receptor TLR9 were not significantly more susceptible to *S*. *pyogenes* infection than control mice (Fig. 3C). The requirement for both TLR2 and Unc93b1 pathways in mouse defense against *S*. *pyogenes* implied that a strong activation of innate responses is favorable for the host. In conclusion, the findings demonstrate that a complete dual TLR activation of innate immune cells is of critical importance for defense against *S*. *pyogenes* in mice. The data also provide the strongest evidence yet for a crucial contribution of pathogen-derived RNA recognition to the initiation of protective host response.

### Human macrophages sense *S*. *pyogenes* through TLR2 but they also employ TLR2-independent pathways that differ from TLR13-mediated recognition

*S*. *pyogenes* is not considered to be pathogenic in mice outside the laboratory [32,33]. Our data demonstrate that mice launch protective immune responses by recognizing *S*. *pyogenes* RNA by TLR13. TLR13 occurs in mice but not humans raising the question whether *S*. *pyogenes* RNA is recognized in human innate immune cells by a different TLR.

**Fig 4:**
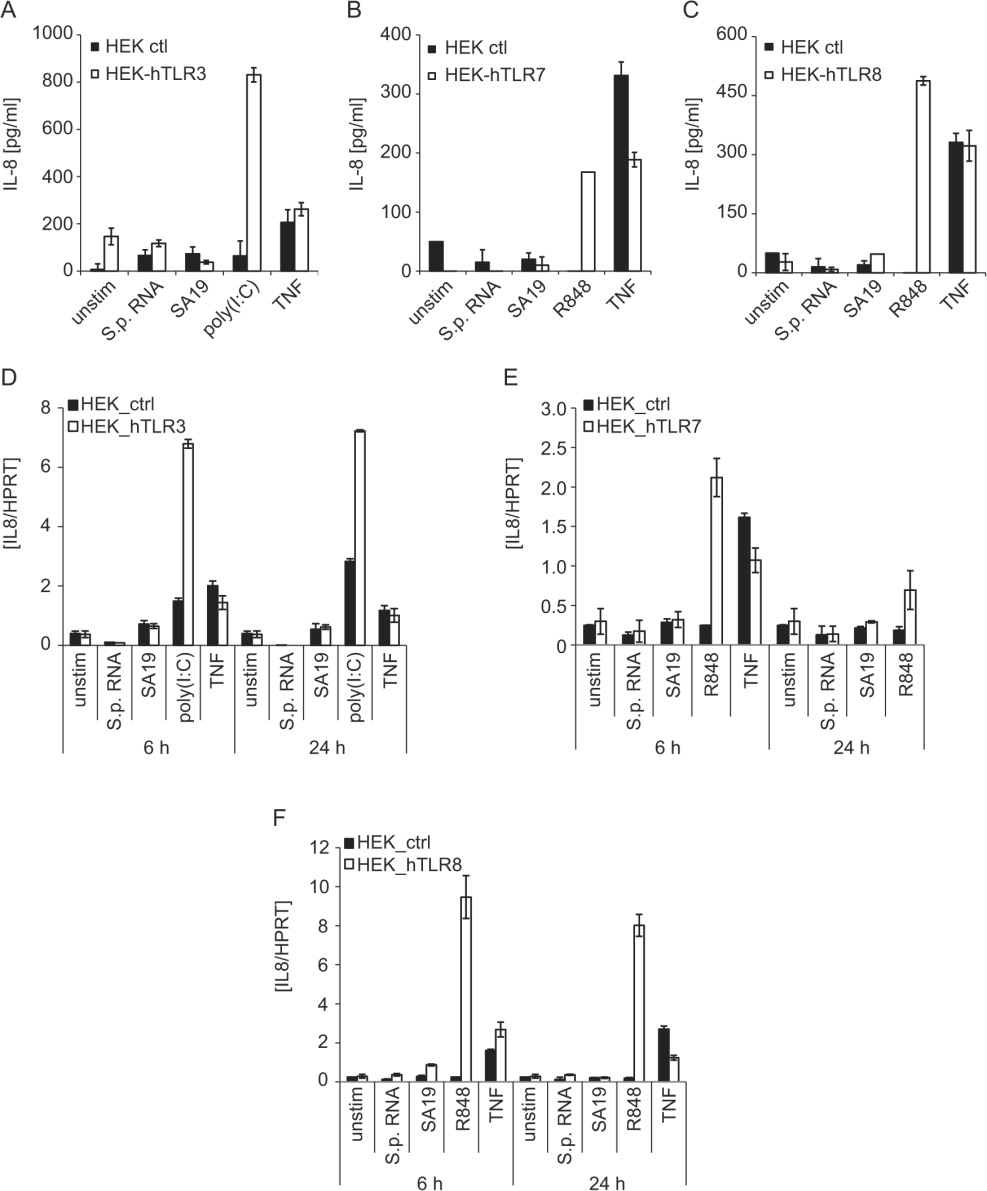
Human RNA-recognizing TLRs are not capable of sensing *S*. *pyogenes* RNA. (A, D) HEK293 stably expressing human TLR3 (HEK293-hTLR3) or control HEK293 cells (HEK ctl) were transfected with the TLR3 ligand poly(I:C) (5 μg), the Tlr13 ligand oligoribonucleotide SA19 (5 μg), *S*. *pyogenes* RNA (5 μg) using DOTAP or stimulated with human TNF (10 ng/ml). IL-8 release was measured in supernatants 24 h post stimulation by ELISA (A) or the levels of *IL8* mRNA were determined by qRT-PCR (normalized to *HPRT*) (D). Mean values ± SD are shown (n ≥ 3). (B, C, E, F) HEK293XL cells stably expressing human TLR7 (B, E) or TLR8 (C, F) or control HEK293XL cells were transfected with SA19 (5 μg), *S*. *pyogenes* RNA (5 μg) or stimulated with the TLR7 and TLR8 ligand R848 (5 μg/ml) or human TNF (10 ng/ml). IL-8 release was measured in supernatants 24 h post stimulation by ELISA (B, C) or the levels of *IL8* mRNA were determined by qRT-PCR (normalized to *HPRT*) (E, F). Mean values ± SD are shown (n ≥ 3).

To address *S*. *pyogenes* RNA recognition by human cells, we employed HEK293 cells stably expressing human TLR3, TLR7 or TLR8, the only known RNA-recognizing TLRs in humans [34]. *S*. *pyogenes* RNA and the reported TLR13 ligand oligoribonucleotide SA19 [16,17] were not stimulatory, regardless of the TLR tested, as assessed by IL-8 protein and mRNA expression (Fig. 4). The TLR3 ligand polyinosinic:polycytidylic acid (poly(I:C)), as well as the TLR7 and TLR8 agonist R848, elicited responses in HEK293 cells expressing the cognate TLR, and as expected, TNF stimulated HEK293 cells independently of TLRs (Fig. 4). In conclusion, these results show that none of the RNA-recognizing human TLRs is capable of substituting for the missing TLR13.

**Fig 5:**
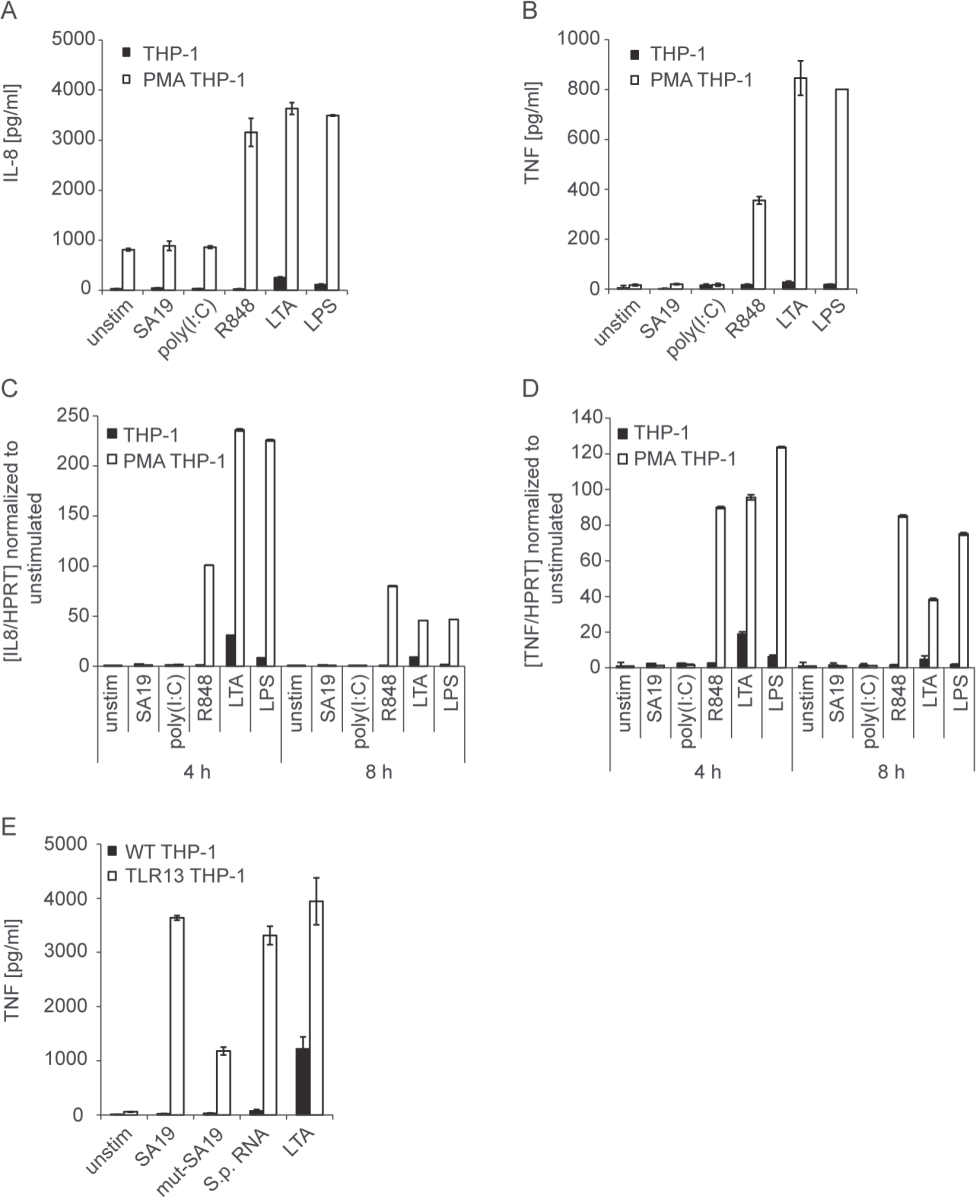
Macrophage-like differentiated THP-1 cells respond to endosomal TLR ligands but not to the canonical TLR13 ligand. (A, B) THP-1 cells were either treated with 10 nM PMA or left untreated (as described in Material and Methods) before stimulation with R848 (5 μg/ml), LTA (5 μg/ml), LPS (10 ng/ml) or DOTAP-mediated delivery of poly(I:C) (5 μg). Supernatants were collected after 8 h and IL-8 (A) or TNF (B) release was determined by ELISA. Error bars represent SDs (n≥3). (C, D) RNA was isolated from differentiated or undifferentiated THP-1 cells treated as described in (A) and (B) for 4 or 8 h. *IL8* (C) and *TNF* (D) mRNA levels were determined by qRT-PCR (normalized to *HPRT*). Mean values ± SD are shown (n ≥ 3). (E) THP-1 cells stably expressing TLR13 (TLR13 THP-1) were stimulated with TLR13 ligand SA19, poor TLR13 ligand (mut-SA19), *S*. *pyogenes* RNA or LTA for 4 h. TNF was determined in supernatants by ELISA. Error bars in represent SDs (n≥3).

**Fig 6:**
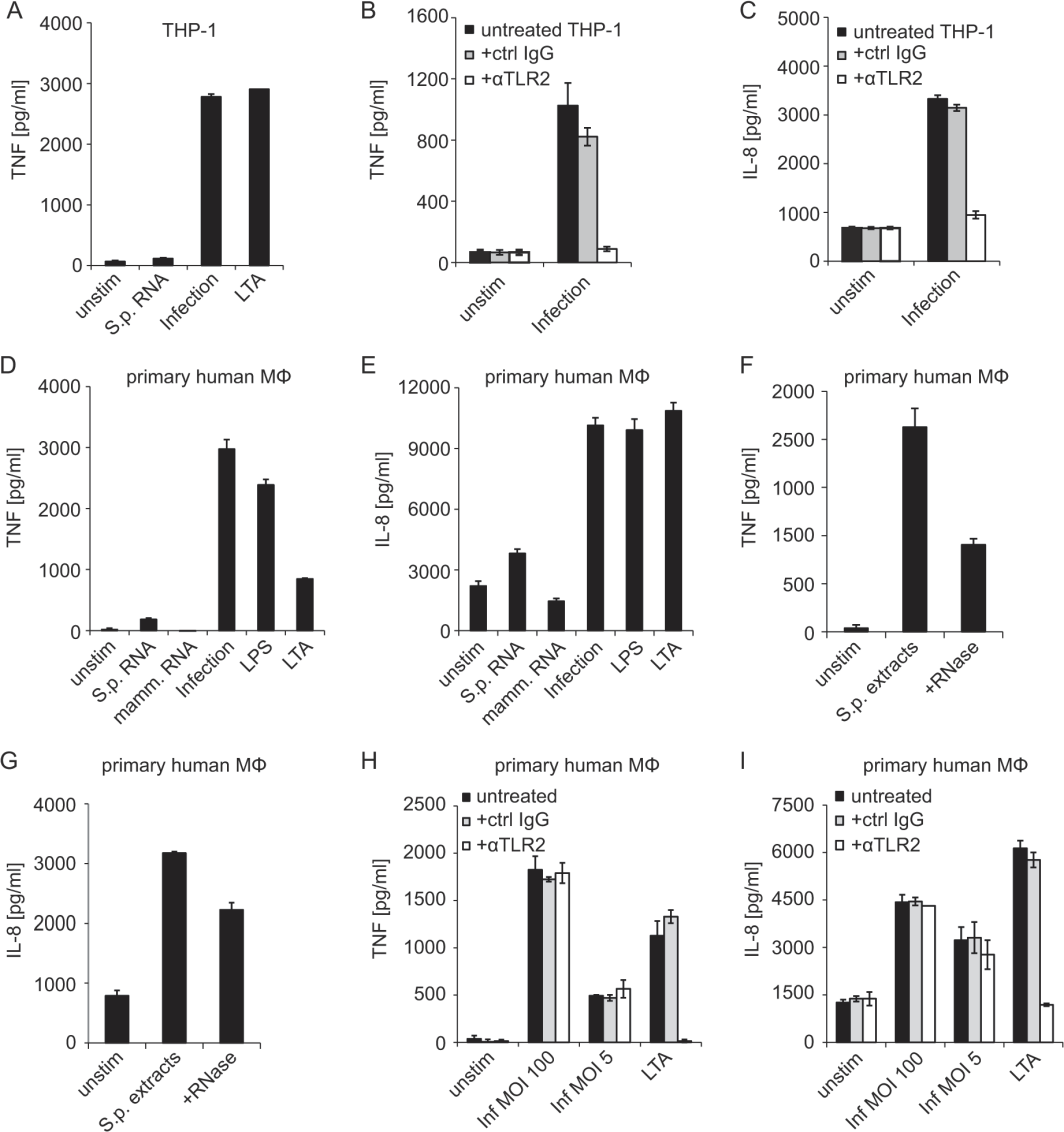
Human macrophage-like cells respond to *S*. *pyogenes* infection by TLR2-dependent signaling but fail to recognize *S*. *pyogenes* RNA. (A) PMA-differentiated THP-1 macrophages were stimulated with *S*. *pyogenes* RNA (5 μg, using DOTAP), LTA (5 μg/ml), infected with *S*. *pyogenes* (MOI = 100) or left untreated. Supernatants were collected 6 h after treatment and TNF release was measured by ELSA. (B, C) PMA-differentiated THP-1 were incubated with an anti-TLR2 (αTLR2) or control IgG antibody for 30 min or left untreated prior to infection with *S*. *pyogenes* (MOI = 100), supernatants were collected 6 h post infection and TNF (B) or IL-8 (C) release was measured by ELISA. (D, E) Primary human macrophages were either transfected with 5 μg *S*. *pyogenes* or mammalian RNA using DOTAP, infected with *S*. *pyogenes* (MOI = 100), stimulated with LTA (5 μg/ml) or LPS (10 ng/ml) or left untreated. Supernatants were collected after 24 h and TNF (D) or IL-8 (E) release was measured by using ELSA. (F, G) Primary human macrophages were stimulated with *S*. *pyogenes* extracts, *S*. *pyogenes* extracts treated with RNase (+RNase), or left unstimulated. Supernatants were collected after 6 h and TNF (F) or IL-8 (G) release was measured by ELSA. (H, I) Primary human macrophages were incubated with an anti-TLR2 (αTLR2) or control IgG antibody for 30 min or left untreated prior to infection with *S*. *pyogenes* (MOI = 100 and MOI = 5) or stimulation with LTS. Supernatants were collected 6 h post infection and TNF (H) or IL-8 (I) release was measured by ELISA. Error bars in all panels represent SDs (n≥3).

To explore the recognition of live *S*. *pyogenes* by human innate immune cells, we employed the human monocytic cell line THP-1. THP-1 cells respond only poorly to TLR ligands, unless differentiated into more mature macrophage-like cells [35]. THP-1 cells differentiated into macrophages using phorbol-12-myristate-13-acetate (PMA) exhibited increased responsiveness to LPS, lipoteichoic acid (LTA) and R848, as compared to undifferentiated THP-1 cells (Fig. 5A-D). Importantly, TNF and IL-8 measurements revealed that PMA-differentiated THP-1 cells were responding neither to the canonical TLR13 ligand oligoribonucleotide SA19 [16,17] nor to *S*. *pyogenes* RNA (Fig. 5A-D and 6A). To test whether expression of murine TLR13 renders THP-1 cells responsive to bacterial RNA we generated THP-1 cells stably expressing TLR13. Stimulation of these cells with oligoribonucleotide SA19 or *S*. *pyogenes* RNA resulted in a strong TNF induction whereas stimulation with a poor TLR13 ligand (mutated oligoribonucleotide SA19, mut-SA19 [16,17]) caused a low TNF induction. These data demonstrate that human THP-1 macrophages were not able to sense bacterial RNA corroborating the observed inability of HEK293 cells expressing human TLR3, TLR7 or TLR8, to respond to bacterial RNA. However, exogenous TLR13 allows THP-1 cells to sense bacterial RNA in a similar way as the endogenous TLR13 in mouse cells.

LTA treatment of THP-1 cells resulted in TNF induction similar to infection with *S*. *pyogenes* (Fig. 6A) suggesting that *S*. *pyogenes* was recognized through TLR2. After blocking TLR2, THP-1 cells were no longer responding to infection with *S*. *pyogenes* (Fig. 6B-C) indicating that TLR2 was the key receptor in these cells. We then examined TNF and IL-8 production by primary human macrophages treated with *S*. *pyogenes* RNA or live bacteria. Similar to THP-1 cells, primary human macrophages were barely responding to *S*. *pyogenes* RNA but reacted well to infection (Fig. 6D-E). Interestingly, DOTAP-mediated stimulation of primary macrophages with bacterial extracts revealed that these cells were activated by bacterial components delivered into the cells (Fig. 6F-G). Extracts treated with RNase were also capable of inducing TNF and IL-8 although the activity was lower than that of untreated extract (Fig. 6F-G). Thus, experiments using isolated *S*. *pyogenes* RNA (Fig. 6D-E) and bacterial extracts (Fig. 6F-G) indicated that primary human macrophages were stimulated by bacterial products delivered into the cells, with bacterial RNA contributing to the stimulatory activity. However, the delivery of purified *S*. *pyogenes* RNA was not capable of activating primary human macrophages (Fig. 6D-E), which is in marked contrast to mouse cells (Fig. 1H-I). Finally, infection of primary human macrophages with *S*. *pyogenes* in the presence of a TLR2-blocking antibody revealed that the cells were responding under conditions of TLR2 inhibition (Fig. 6H-I). Reduction of MOI from 100 to 5 did not change the inability of the TLR2-blocking antibody to prevent TNF and IL-8 production although the antibody efficiently inhibited responses to LTA (Fig. 6H-I).

Together, these data demonstrate that primary human macrophages differ from THP-1 cells in that they are able to sense *S*. *pyogenes* independently of TLR2. Neither THP-1 nor primary human macrophages are able to respond to bacterial RNA although bacterial RNA might contribute to responses of primary human macrophages if delivered together with bacterial extracts.

### TLR13 is absent from most mammals but occurs in other vertebrates, insects, Annelida and plants

**Fig 7:**
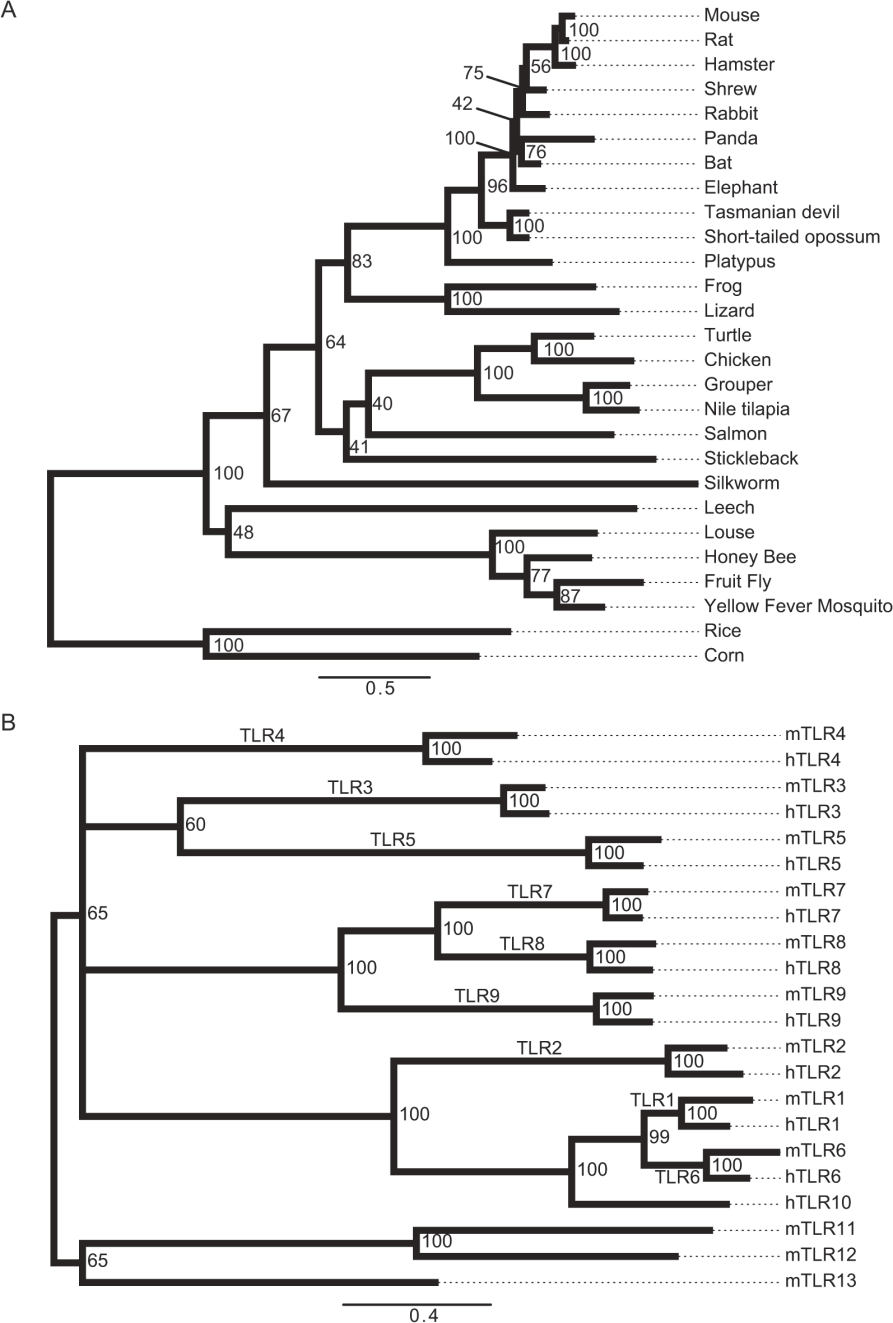
Phylogenetic analysis of TLR13 in species and of the TLR repertoires in humans and mice. (A) The phylogenetic tree displays a choice of species where we identified an ortholog to TLR13 using the OMA browser and NCBI Blast search. The hits were confirmed with FACT to test whether the predicted orthologs have similar feature architecture as the query protein. Thus, the orthologs are firstly similar in sequence and secondly similar in their feature architecture. Numbers at the branch points indicate bootstrap values. (B) The phylogenetic tree displays evolutionary relationship of human and mouse TLR proteins. The tree was constructed as described in (A). Note that if a particular TLR is found in both species then both orthologs exhibit very close evolutionary relationship (bootstrap value 100). The nucleic acid-sensing TLRs do not cluster in one branch: TLR7, TLR8 and TLR9 are found in a different branch than TLR3, and the mouse-specific TLR13 clusters yet in another branch together non-nucleic acid sensors TLR11 and TLR12. Numbers at the branch points indicate bootstrap values (cut-off > 50).

To obtain a more comprehensive picture of the presence of TLR13 and the bacterial rRNA recognition pathway various organisms we performed a phylogenetic analysis of the predicted TLR13 protein sequences in all kingdoms (Fig. 7A and S1 Table). TLR13 is absent in primates and most other mammals. TLR13 occurs in mice and rats, but is missing in other rodents including guinea pig and squirrel. However, TLR13 is found in non-mammalian vertebrates as well as in other kingdoms including insects, Annelids and plants. The mouse and rat TLR13 proteins are highly conserved but distant from TLR13 found in other non-mammalian species (Fig. 7A). Thus, TLR13 appears to have been lost in primates and most other mammals, except for e.g. mice and rats. We directly compared the entire TLR repertoires of mice and humans by generating a TLR tree for these two species (Fig. 7B). The tree revealed that TLR11, TLR12 and TLR13, which occur in mice but not humans, are located in one branch. Interestingly, these three endosomal TLRs do not group together with other known endosomal TLRs, most notably the nucleic acid-sensing TLR7, TLR8 and TLR9, which cluster in a separate branch. The remaining nucleic acid-sensing TLR3 groups with the flagellin-recognizing TLR5 in yet another branch, however this branch is only weakly supported (bootstrap value 60%). The distribution of nucleic sensing TLRs in three different branches indicates that the ability of sensing nucleic acids has been acquired before the final diversification of TLRs.

## Discussion

Microbes colonizing surface tissues of complex multicellular organisms consist of harmless species as well as of species capable of converting into invading pathogens, when the surface barrier function is disabled. In this study we establish that the human pathogen *S*. *pyogenes*, which asymptomatically colonizes one-third of the human population and as such regarded as commensal microbe [36,37], elicits innate immune responses by two recognition modes in mice: 1) Host cell surface-localized sensing of *S*. *pyogenes* through TLR2; 2) Endosome-localized sensing of *S*. *pyogenes* rRNA through TLR13 upon phagocytosis of the pathogen. Activation of both pathways is required for successful defense in mice showing that only a dual TLR stimulation launches an adequate immune response. Our results further reveal that the recognition of *S*. *pyogenes* RNA by the TLR system is missing in human cells. We find that TLR13 occurs in mammals only in exceptional cases, with mice and rats being prominent examples. Notably, *S*. *pyogenes* is not a natural pathogen in these two mammalian species [32,33].

Previous studies revealed a critical role of TLR-mediated innate immune response in protecting mice against *S*. *pyogenes*: mice deficient in MyD88 exhibited decreased survival in a subcutaneous infection model [14], the model we employed in this study. IL-1β, which also signals via MyD88, has been proposed to be dispensable for host defense as mice deficient in the Nlrp3 inflammasome did not exhibit increased susceptibility to *S*. *pyogenes* in an intraperitoneal infection model [38]. Despite the well-defined role of MyD88 in host protection, in vitro infection experiments have so far failed to identify an essential role for any TLR tested [13,15]. In this study, we observe that only the absence of both TLR2 and Unc93b1 abolishes cytokine production. These TLR pathways are largely redundant in vitro explaining the lack of measurable effects of TLR2 deficiency described in previous reports [13,15]. The less redundant response in cDCs compared to BMDMs may result from differences in signal processing modules in these two cell types. Indeed, *S*. *pyogenes* RNA induces IFN-β production in cDCs but not in BMDMs [21]. Similar to TLR2/Unc93b1 double-deficient cells, *S*. *pyogenes*-induced cytokine production is abolished also in TLR13-deficient cells under conditions of blocked TLR2 signaling revealing the key functions of TLR2 and TLR13 in *S*. *pyogenes* sensing. TLR9 has been reported to enhance survival of mice in an intraperitoneal model of *S*. *pyogenes* infection [39]. Thus, in that infection model TLR9 may contribute to mouse protection by sensing *S*. *pyogenes* DNA by cells other than BMDMs and cDCs, or indirectly, by its known ability to sense self-DNA liberated from dead cells [40].

TLR13 recognizes with a remarkable specificity a highly conserved bacterial rRNA sequence [16,17]. Given the ubiquitous presence of the TLR13 ligand it is surprising that the contribution of this receptor to immune responses against bacteria has remained unknown until very recently. The major reason for the long unrecognized function of TLR13 is the dominant role of other TLRs, most notably TLR2 and TLR4, in sensing of many bacteria. In contrast to this, sensing of *S*. *pyogenes* occurs through a partially redundant engagement of TLR2 and TLR13 in mouse cells. It is at present unclear why stimulation of TLR2 by *S*. *pyogenes* peptidoglycan and LTA, which in purified form are capable of triggering TLR2 [41], does not play an equally dominant role in recognition as reported for other Gram-positive pathogens including *Staphylococcus aureus* or *Listeria monocytogenes* [42,43]. *S*. *pyogenes* has no robust life cycle once internalized by host cells which employ both phagolysosomal killing and autophagy to eradicate this pathogen [44]. Thus, it is conceivable that the efficient multilayered processing of *S*. *pyogenes* within host cells allows a better exposure of TLR13 to bacterial rRNA when compared to other Gram-positive bacteria, particularly those capable of intracellular growth. A similarly important role of TLR13 is likely to be common in the recognition of other streptococci as shown e.g. by TLR13-dependent recognition of *S*. *pneumoniae* RNA and by Unc93b1-dependent recognition of Group B Streptococcus (GBS) RNA [16,45]. Notably, a recent study revealed that GBS rRNA but not rRNA devoid of the TLR13 recognition sequence was able to stimulate mouse macrophages implying that this pathogen is sensed through TLR13 [46].

The lack of TLR13 in humans is intriguing given the absolute level of conservation of the TLR13 ligand in both Gram-positive and -negative bacteria [16,17]. Our findings establish that human RNA-recognizing TLRs cannot compensate for the lack of TLR13. Consistently, human innate immune cells do not respond to *S*. *pyogenes* RNA by production of TNF and IL-8. Although human THP-1 macrophages employ solely TLR2 for recognition of *S*. *pyogenes*, this simple mode of sensing is not operational in primary human macrophages: Primary cells recognize products present in bacterial extracts delivered into the cells. Bacterial RNA, if delivered in combination with other components of the bacterial extract, might contribute to this way of host cell stimulation. Future studies should elucidate the nature of intracellular recognition receptors involved in recognition of *S*. *pyogenes* in primary human cells. Such receptors may be different from TLRs since a recent study revealed that the human but not the mouse NLRP3 inflammasome is activated by all three bacterial RNA species (mRNA, rRNA and tRNA) [47].

The lack of TLR13 is fully penetrant in primates and widespread among mammals. The presence of predicted TLR13 in non-mammalian vertebrates, insects and plants indicates that primates have lost this TLR early in their evolution, whereas mice and rats preserved the gene. The selective maintenance of TLR13 in a very limited number of mammalian species poses the elementary question of the evolutionary pressure imposed on most mammals to lose TLR13, and of the evolutionary advantage gained in mice and rats by keeping this gene. Primates might have eliminated TLR13 in order to limit the lifelong burden of continuous sensing of rRNA derived from e.g. environmental microbiota or to increase tolerance to bacterial infections [48,49]. Alternatively, the existence of pathways not present in mice, for example a more promiscuous human NLRP3 inflammasome [47], might provide human cells with immune mechanisms, which can compensate for the evolutionary loss of TLR13. The resistance of mice and the susceptibility of humans to *S*. *pyogenes* infection involves specific differences in host-pathogen interactions including components of the complement system, antimicrobial peptides and activation of T cells by streptococcal superantigens, but the full complexity of the divergences is not well understood [8,50,51]. Our study suggests that the differences in recognition of *S*. *pyogenes* by mouse compared to human cells might play an important role in susceptibility to infection.

The data presented here establish *S*. *pyogenes* RNA as critical player in successful immune defense of mice and uncover a previously unrecognized role of TLR2 in mouse and man in recognition of this pathogen.

## Supporting Information

S1 Table Tlr13 is missing in primates but occurs in mice and rarely in other mammals while it can be found in non-mammalian vertebrates, insects, Annelida and plants. Tlr13 orthologs were searched for and identified using the OMA browser (light blue) or a standard NCBI Blast search against non-redundant protein sequences (dark blue). Putative hits were tested for occurrence of similar feature architecture as in the query protein, i.e. mouse Tlr13, using FACT (Feature Architecture Comparison Tool; http://www.cibiv.at/FACT). Hits exhibiting similar protein architecture as mouse Tlr13 were defined as predicted orthologs and are depicted in light blue (hits obtained using the OMA browser) or dark blue (hits obtained using NCBI Blast). Shown is a choice of mammalian species as well as Tlr13-containing representatives from other kingdomes.
(PDF)
