# Supplementary material for: Innate Immune Response to Streptococcus pyogenes Depends on the Combined Activation of TLR13 and TLR2
Source: PLoS One. 2015 Mar 10;10(3):e0119727. doi: 10.1371/journal.pone.0119727 (PMC4355416; doi:10.1371/journal.pone.0119727)
Supplement: S1 Table — Tlr13 orthologs were searched for and identified using the OMA browser (light blue) or a standard NCBI Blast search against non-redundant protein sequences (dark blue). Putative hits were tested for occurrence of similar feature architecture as in the query protein, i.e. mouse Tlr13, using FACT (Feature Architecture Comparison Tool; http://www.cibiv.at/FACT). Hits exhibiting similar protein architecture as mouse Tlr13 were defined as predicted orthologs and are depicted in light blue (hits obtained using the OMA browser) or dark blue (hits obtained using NCBI Blast). Shown is a choice of mammalian species as well as Tlr13-containing representatives from other kingdomes. (PDF) [file pone.0119727.s001.pdf]

Table S1. Tlr13 is missing in primates but occurs in mice and rarely in other mammals while it can be found in non-mammalian vertebrates, insects, Annelida and plants.

The table lists a choice of mammalian species in which Tlr13 was searched for, as well as representative vertebrate, insect, Annelida and plant species containing Tlr13. The Tlr13 orthologs were searched for and identified using the OMA browser (light blue) or a standard NCBI Blast search against non-redundant protein sequences (dark blue). Putative hits were tested for occurrence of similar feature architecture as in the query protein, i.e. mouse Tlr13, using FACT (Feature Architecture Comparison Tool; http://www.cibiv.at/FACT). Hits exhibiting similar protein architecture as mouse Tlr13 were defined as predicted orthologs and are depicted in light blue (hits obtained using the OMA browser) or dark blue (hits obtained using NCBI Blast).

**Species**	**Common name**	**Tlr13**	**Order**	**Class**	**Kingdom**
Rattus norvegicus	brown or norway rat	YES	Rodentia	Mammalia	Animalia
Mus musculus	house mouse	YES	Rodentia	Mammalia	Animalia
Spermophilus tridecemlineatus	thirteen-lined ground squirrel	NO	Rodentia	Mammalia	Animalia
Cavia porcellus	guinea pig	NO	Rodentia	Mammalia	Animalia
Dipodomys ordii	ord's kangaroo rat	NO	Rodentia	Mammalia	Animalia
Cricetulus griseus	Chinese hamster	YES	Rodentia	Mammalia	Animalia
Oryctolagus cuniculus	european rabbit	YES	Lagomorpha	Mammalia	Animalia
Homo sapiens	human	NO	Primates	Mammalia	Animalia
Gorilla gorilla	western gorilla	NO	Primates	Mammalia	Animalia
Pan troglodytes	common chimpanzee	NO	Primates	Mammalia	Animalia
Pongo abelii	Sumatran orangutan	NO	Primates	Mammalia	Animalia
Nomascus leucogenys	white-cheeked gibbon	NO	Primates	Mammalia	Animalia
Macaca mulatta	rhesus macaque	NO	Primates	Mammalia	Animalia
Callithrix jacchus	common marmoset	NO	Primates	Mammalia	Animalia
Tupaia chinensis	chinese tree shrew	YES	Scandentia	Mammalia	Animalia
Loxodonta africana	african bush elephant	YES	Proboscidea	Mammalia	Animalia
Equus caballus	horse	NO	Perissodactyla	Mammalia	Animalia
Canis familiaris	domestic dog	NO	Carnivora	Mammalia	Animalia
Ailuropoda melanoleuca	giant panda	YES	Carnivora	Mammalia	Animalia
Felis catus	domestic cat	NO	Carnivora	Mammalia	Animalia
Bos taurus	european cattle	NO	Artiodactyla	Mammalia	Animalia
Sus scrofa	wild pig	NO	Artiodactyla	Mammalia	Animalia
Myotis lucifugus	little brown bat	YES	Chiroptera	Mammalia	Animalia
Dasypus novemcinctus	nine-banded armadillo	NO	Cingulata	Mammalia	Animalia
Sarcophilus harrisii	Tasmanian devil	YES	Dasyuromorphia	Mammalia	Animalia
Macropus eugenii	tammar wallaby	NO	Diprotodontia	Mammalia	Animalia
Monodelphis domestica	gray short-tailed opossum	YES	Didelphimorphia	Mammalia	Animalia
Ornithorhynchus anatinus	platypus	YES	Monotremata	Mammalia	Animalia
Anolis carolinensis	american cameleon	YES	Squamata	Reptilia	Animalia
Chelonia mydas	green sea turtle	YES	Testudines	Reptilia	Animalia
Gallus gallus	red junglefowl	YES	Galliformes	Aves	Animalia
Xenopus tropicalis	western clawed frog	YES	Anura	Amphibia	Animalia
Gasterosteus aculeatus	three-spined stickleback	YES	Gasterosteiformes	Actinopterygii	Animalia
Salmo salar	atlantic salmon	YES	Salmoniformes	Actinopterygii	Animalia
Epinephelus coioides	orange-spotted grouper	YES	Perciformes	Actinopterygii	Animalia
Oreochromis niloticus	nile tilapia	YES	Perciformes	Actinopterygii	Animalia
Drosophila melanogaster	common fruit fly	YES	Diptera	Insecta	Animalia
Aedes aegypti	yellow fever mosquito	YES	Diptera	Insecta	Animalia
Apis mellifera	western honey bee	YES	Hymenoptera	Insecta	Animalia
Pediculus humanus	body or head louse	YES	Phthiraptera	Insecta	Animalia
Helobdella robusta	californian leech	YES	Rhynchobdellida	Clitellata	Animalia
Oryza sativa	rice	YES	Poales	Monocots	Plantae
Zea mays	corn	YES	Poales	Monocots	Plantae
